# Prevalence of high-risk human papillomavirus cervical infection in female kidney graft recipients: an observational study

**DOI:** 10.1186/1743-422X-9-117

**Published:** 2012-06-18

**Authors:** Bronislawa Pietrzak, Natalia Mazanowska, Alicja M Ekiel, Magdalena Durlik, Gayane Martirosian, Mirosław Wielgos, Pawel Kaminski

**Affiliations:** 1 First Department of Obstetrics and Gynecology, Medical University of Warsaw, Pl. Starynkiewicza 1/3, 02-015, Warszawa, Poland; 2 Department of Medical Microbiology, Medical University of Silesia, ul. Medykow 18, 40-752, Katowice, Poland; 3 Department of Transplantation Medicine and Nephrology, Transplantation Institute, Medical University of Warsaw, ul. Lindleya 4, 02-015, Warszawa, Poland; 4 Department of Histology and Embryology, Medical University of Warsaw, ul. Chalubinskiego 4, 02-004, Warszawa, Poland

**Keywords:** Renal transplantation, HPV mRNA, HR-HPV, Immunosuppressive therapy, Cervical intraepithelial neoplasia

## Abstract

**Background:**

Immunosuppressive therapy protects the transplanted organ but predisposes the recipient to chronic infections and malignancies. Transplant patients are at risk of cervical intraepithelial neoplasia (CIN) and cervical cancer resulting from an impaired immune response in the case of primary infection or of reactivation of a latent infection with human papillomavirus of high oncogenic potential (HR-HPV).

**Methods:**

The aim of this study was to assess the prevalence of HR-HPV cervical infections and CIN in 60 female kidney graft recipients of reproductive age in comparison to that in healthy controls. Cervical swabs were analyzed for the presence of HR-HPV DNA. HR-HPV-positive women remained under strict observation and were re-examined after 24 months for the presence of transforming HR-HPV infection by testing for HR-HPV E6/E7 mRNA. All the HR-HPV-positive patients were scheduled for further diagnostic tests including exfoliative cytology, colposcopy and cervical biopsy.

**Results:**

The prevalence of HR-HPV did not differ significantly between the study group and the healthy controls (18% vs 25%, p = 0.37). There was no correlation between HR-HPV presence and the immunosuppresive regimen, underlying disease, graft function or time interval from transplantation. A higher prevalence of HR-HPV was observed in females who had had ≥2 sexual partners in the past. Among HR-HPV-positive patients, two cases of CIN2+ were diagnosed in each group. In the course of follow-up, transforming HR-HPV infections were detected in two kidney recipients and in one healthy female. Histologic examination confirmed another two cases of CIN2+ developing in the cervical canal.

**Conclusions:**

Female kidney graft recipients of reproductive age are as exposed to HR-HPV infection as are healthy individuals. Tests detecting the presence of HR-HPV E6/E7 mRNA offer a novel diagnostic opportunity in those patients, especially in those cases where lesions have developed in the cervical canal.

## Background

Solid organ recipients receive immunosuppressive therapy to protect the transplanted organ against rejection, which therapy also unfortunately predisposes them to chronic infections and the development of malignancies [1]. According to the literature data renal transplant recipients have a high risk of developing anogenital tract malignancies [2,3]. Carcinoma of the uterine cervix accounts for approximately 3% of all malignancies among transplant patients [4]. Renal transplantation increases the incidence of cervical intraepithelial neoplasia up to 14–16 fold, and that of invasive cervical cancer 3.0–8.6 fold [5-7]. The actual role of immunosuppressive therapy in the development of malignancy remains unclear. In the light of current data, it seems that immunosuppression, while not causing the progression of the carcinoma itself, is mostly linked to the early stages of dysplasia due to the mechanisms responsible for the elimination of high-risk human papillomavirus (HR-HPV) infection being defective [8]. At present there are still no convincing and unanimous HPV screening data from renal transplant recipients. Positive HR-HPV DNA tests are more sensitive than exfoliative cytology in predicting females at risk of developing cervical intraepithelial neoplasia (CIN) or carcinoma of the cervix [9]. However, HR-HPV DNA tests have poor diagnostic specificity. This is due to the very high prevalence of transient HPV infections [10], as opposed to the persistent HPV infections that lead to precancerous and cancerous lesions of the uterine cervix. This is characterized by a shift from a productive to a transforming infection resulting from a switch in viral gene expression. Increased expression of viral oncogenes E6 and E7 prevents the infected cells from terminal differentiation by interfering with cell-cycle regulating proteins p53 and Rb. This leads to the HPV infection being maintained, because infected cells do not undergo apoptosis, but on the contrary experience activation of their pro-proliferative genes [11,12]. The specificity of cervical screening might therefore be improved by the detection of biomarkers distinctive of transforming infection. Obviously, HPV DNA testing alone cannot discriminate between transient and transforming infections. However, the expression of viral oncogenes E6 and E7 is regarded as a signal of potential progression to invasive cervical cancer [12]. Therefore it was proposed that the detection of HPV E6 and E7 mRNAs could serve to identify patients with an elevated risk of CIN and cervical cancer [13]. The study was designed to investigate the prevalence of HR-HPV DNA in cervical specimens, as well as the prevalence of transforming HPV infection among HPV-positive females, by detecting HPV E6 and E7 mRNAs. It also aimed to assess the clinical value of these tests in the cervical screening of kidney graft recipients in comparison to that of healthy women.

## Results

HR-HPV infection was diagnosed in 11/60 kidney graft recipients and 15/60 healthy women, indicating a comparable infection rate (p = 0.37) in the two groups. HR-HPV was detected significantly more often in females having had >2 sexual partners in the past. There was, however, no correlation observed between the presence of HPV in kidney graft recipients and serum creatinine concentration, age, body mass index (BMI), immunosuppressive therapy regimen and underlying or coexisting diseases.

On initial screening, among the HR-HPV DNA-positive women abnormal Pap smear results were detected in two kidney graft recipients (2 cases of HSIL) and two healthy women (LSIL and HSIL). After colposcopy-guided cervical biopsy, in all cases CIN2+ was diagnosed and the patients were scheduled for further treatment. Three of them were offered loop electrosurgical excision procedure (LEEP) and histological examination confirmed that the lesions were excised with negative margins. In one case (a transplant patient) extirpation of the uterus was offered because of concomitant uterine fibroids accompanied by excessive menstrual bleeding, leading to severe anemia that was resistant to hormonal therapy.

On follow-up after twenty four months, it was established that the rate of clearance of HR-HPV infection of the uterine cervix was similar in both groups, reaching 81,8% in the study group. In the control group, all women but one (93%) were HR-HPV negative at the time of follow-up (p = ns).

Among renal transplant recipients, a persistent infection with HR-HPV was confirmed in two cases. Both women had a positive E6/E7 mRNA test and were scheduled for additional investigations. After colposcopy-guided cervical biopsy, CIN3 was diagnosed in one case and the patient was scheduled for further treatment (LEEP excision procedure). The histological evaluation of specimens from the cervical canal confirmed the previous diagnosis of CIN3. Significantly, this patient displayed a normal cervical cytology, even though the pathologist had been informed about the presence of HPV E6/E7 mRNA. The other transplant patient had a normal Pap smear and innocuous colposcopy finding, but nevertheless qualified for cervical biopsy and canal curettage. Histological examination revealed no pathological lesions in collected tissue samples.

Similarly, HPV E6/E7 mRNA-positive females from the control group underwent additional examinations. Repeated Pap smear results revealed HSIL. Colposcopy and subsequent biopsies led to the diagnosis of CIN3 in tissues obtained during cervical canal curettage. This patient was offered LEEP and histological examination confirmed that CIN3 in the cervical canal had been excised with negative margins.

## Discussion

There are relatively few publications dealing with HR-HPV infection in populations of transplant recipients. The incidence of HR-HPV infection as well as abnormal Pap smear results is reported to be quite high in recipients, reaching 59% according to some studies [14-16]). However, other data suggest a low prevalence of HPV infections in kidney graft recipients, similar to that of the general population [17]. In our study the prevalence of HR-HPV infections in both groups was similar, with the prevalence in immunocompromised women tending to be slightly lower than that in healthy controls (18,6% vs 25%, p = ns). A recently published Italian study by Origoni *et al* suggested that transplant recipients receiving long-term immunosuppression may not be more at risk of HPV infection and CIN than healthy controls [18], which is in agreement with our results. However, the mean recipient age in the above mentioned Italian study approached 38 years, and in our study 37 years, whereas it is well established that younger women are especially prone to HPV infection [19]. Morrison *et al* suggested that HR-HPV infection was relatively rarely encountered among older renal graft recipients with normal Pap smear results. This applied in particular to monogamous or sexually inactive patients. Therefore it was recommended that education about avoiding high-risk sexual relations should be of prime importance among kidney graft recipients [17]. Our results support the above mentioned recommendation. During the analysis of the correlation between HR-HPV infection and the number of lifetime sexual partners, it was noted that the incidence of HPV was slightly higher among patients from both groups that had had more than one sexual partner in their lifetime; the difference was however statistically insignificant. The kidney recipients also had a significantly higher incidence of having had only one lifetime sexual partner, which may explain the relatively low incidence of cervical HPV infection in our cohort.

Testing for E6/E7 mRNA offers a novel approach in cervical screening. There are published reports pointing out that the introduction of E6/E7 HPV mRNA testing may improve the diagnostic accuracy in HR-HPV-positive women [20]. Benevolo *et al* observed that, in a group of 408 HR-HPV-positive patients with normal Pap smears (regarded as ≤ HSIL), positive HPV mRNA results were significantly more often associated with CIN ≥ 2 than with CIN ≤ 2 lesions. Therefore the authors concluded that testing for E6/E7 HPV mRNA may have a diagnostic and potentially prognostic role in the management of HR-HPV(+), HSIL(−) patients [21].

There is strong evidence that testing for HPV E6/E7 mRNA is more specific than testing for HPV DNA, but the sensitivity of the test may be unsatisfactory for clinical purposes. Benevolo *et al* conducted another study on a group of 1201 participants and concluded that E6/E7 mRNA testing was more efficient than cytology for the triage of HPV DNA-positive women. However, the sensitivity for CIN2+ was relatively low (83% in cases of ASCUS, LSIL 62% and HSIL 67%). That fact means that HPV DNA-positive women, in spite of the negative E6/E7 mRNA test result, would nevertheless require strict follow-up [22]. In our study all the HR-HPV-positive patients, except for three females with transforming infections confirmed by means of the E6/E7 mRNA test, cleared the cervical infection within 24 months of follow-up.

Norwegian authors [23] have raised the issue of specificity of the test, around 30% of their patients having a positive HPV E6/E7 mRNA test result with no lesions being detected by subsequent cervical biopsy. The positive predictive value of HPV E6/E7 mRNA testing in HR-HPV-positive women with minor cervical lesions seen on cytological examination reached 70%. In our study, one of the three HPV E6/E7 mRNA-positive patients—a kidney recipient—had no abnormal tissues detectable in specimens collected during colposcopic-guided cervical biopsy and canal curettage. The explanation proposed by the Norwegian authors is based on the fact that colposcopic biopsy may miss as much as 26-42% of the CIN2+ lesions. Infections with HPV type 18 or 45 with typical localization inside the cervical canal may especially be missed on colposcopy. In fact, the Norwegian authors suggest that the 30% of the mRNA-positive patients with a negative histological confirmation of cervical disease may represent false negative results. Our patient was not scheduled for conization but remains under strict clinical follow-up and will be scheduled for repeat colposcopy and biopsy in 6 months’ time.

## Conclusions

The clinical value of HPV E6/E7 mRNA testing as well as its future position in cervical screening algorithms remains to be established. The possibility of confirming a transforming HPV infection, thereby facilitating the early identification of women at greatest risk of cervical neoplasm development, seems very promising. This applies especially to immunocompromised renal graft recipients, that in the past were often regarded as a high risk group, although the currently published data are inconsistent. Origoni et al found no increase of CIN in population of Italian female renal graft recipients that remained under long-term follow-up [18]. On the other hand a recently published report by Meeuwis *et al* analyzing data from over 1000 female kidney graft recipients confirmed the fivefold increase of risk of cervical cancer in comparison to the general population and emphasized the importance of cervical screening [24]. According to our data, female kidney graft recipients of reproductive age are as exposed to HR-HPV infection as are healthy individuals, which may be regarded as novelty. Our results, despite the small number of HR-HPV-positive females that underwent follow-up, highlight the utility of HPV E6/E7 mRNA detection in those cases where lesions develop in the cervical canal, which often go undetected by exfoliative cytology. Timely detection of cervical dysplasia at the early stages would allow for timely and adequate therapy among these patients. The decrease of cervical cancer morbidity may be achieved in future by means of HPV vaccination, but at present there are insufficient data regarding the efficacy and safety of the available vaccines in populations of iatrogenically immunocompromised females.

## Methods

One hundred twenty women of reproductive age, in outpatient care at the First Department of Obstetrics and Gynecology, Medical University of Warsaw, between 1st October 2007 and 30th September 2008, were evaluated for the study. The study group included 60 kidney graft recipients with normal cervical smears obtained within the previous 12 months. The time interval that had elapsed since the kidney transplantation varied from 7 months to 20 years, with the mean interval being 6.4 ± 4.9 years. Over 76% of the kidney graft recipients (46/60) had undergone transplantation less than 10 years previously. The majority of graft recipients (34/60) were on a regimen of three drugs, consisting mainly of calcineurin inhibitors with corticosteroids and mycophenolan acid precursors. An immunosuppressive regimen of two drugs was prescribed to 24/60 women. The control group consisted of 60 healthy women under routine gynecological care in the outpatient clinic of our Department. The characteristics of both groups are presented in Table 1, and the selection criteria in Table 2.

**Table 1:** Characteristic of the study and control groups

**Characteristic of patients**	**Study group (n = 60)**	**Control group (n = 60)**	**P value**
Age (years)			
min	20	24	
max	48	48	
mean	37,3 ± 7	36,4 ± 7,1	ns
BMI (kg/m2)	23,63 ± 2,43	23,30 ± 3,85	ns
Parity			
min	0	0	
max	5	4	
mean	0,8 ± 0,988	1,08 ± 0,979	
median	0,5	1,0	ns
Number of partners			
1	44	27	P = 0.01
≥2	16	33	

P < 0.05 was considered statistically significant.

**Table 2:** Study inclusion and exclusion criteria

**Inclusion criteria of patients**	**Exclusion criteria**
Age 28–48 years	*Trichomonas vaginalis* infection
regular menstrual cycles	sexual intercourse during previous 4 days
sexually active	antibiotic therapy during past 2 weeks
normal Pap smear obtained within previous 12 months	vaginal spotting
absence of symptoms of genital infection	pregnancy and puerperium
	hormonal contraception
	active malignant disease
	fever in the previous 4 days
	smoking
	lack of informed consent

The cervical swabs were analyzed using the Amplicor HPV Roche Molecular Systems test designed to detect the DNA of thirteen high risk HPV types: 16, 18, 31, 33, 39, 45, 51, 52, 56, 58, 59 and 68. All the participants gave informed consent for enrollment in the study which had been approved by the Bioethical Committee of the Medical University of Warsaw.

After the initial cervical screening described above, a cohort consisting of 15 healthy women and 11 renal transplant recipients showed the presence of HR-HPV DNA in their cervical samples. As follow-up, these patients underwent further investigations. Prompt cervical smears according to the Bethesda/Papanicolau system, as well as colposcopy, were performed in order to plan further treatment if necessary.

The HR-HPV DNA-positive females remained under close observation and cervical swabs were obtained again after 24 months. These were analyzed for the presence of HR-HPV E6/E7 mRNA using the CE-IVD- certified Nucli-Sense EasyQ test (Biomerieux, France), which detected the mRNA of HR-HPV types 16, 18, 31, 33 and 45. All the patients with positive HR-HPV E6/E7 mRNA test results were subjected to further investigations.

All the samples obtained from HPV DNA-positive females after 24 months of follow-up were also analyzed using the CE-IVD-certified Linear Array Genotyping Test (Roche, Switzerland) which detected of DNA of 37 HPV types (6, 11, 16, 18, 26, 31, 33, 35, 39, 40, 42, 45, 51, 52, 53, 54, 55, 56, 58, 59, 61, 62, 64, 66, 67, 68, 69, 70, 71, 72, 73, 81, 82, 83, 84, IS39 and CP6108). This was done in order to exclude the possibility that these females were carrying other persistent cervical HPV infections.

## Abbreviations

ASCUS: Atypical squamous cells of undetermined significance; BMI: Body mass index; CE-IVD: CE mark for *in vitro* diagnostic devices indicating fulfillment of the applicable European Directive; CIN: Cervical intraepithelial neoplasia; CIN1: Minor cervical intraepithelial neoplasia; CIN2: Medium cervical intraepithelial neoplasia; CIN3: Severe cervical intraepithelial neoplasia; CIN2+: CIN2 and 3; HPV: Human papillomavirus; HR-HPV: High risk human papillomavirus; HSIL: High grade squamous intraepithelial lesion; LEEP: Loop electrosurgical excision procedure; LSIL: Low grade squamous intraepithelial lesion.

## Competing interests

The authors declare no conflicts of interests.

## Authors’ contributions

BP designed the study and contributed to the collection and analysis of data and writing of the manuscript. NM contributed to the collection and analysis of data as well as writing of the manuscript. AME, GM, MD, PK and MW contributed to the analysis of data and critical revission of the manuscript. All authors approved the final version of the manuscript.
